# Supplementary material for: From single-sequences to evolutionary trajectories: protein language models capture the evolutionary potential of SARS-CoV-2
Source: Nat Commun. 2026 Feb 19;17:2938. doi: 10.1038/s41467-026-69569-9 (PMC13031934; doi:10.1038/s41467-026-69569-9)
Supplement: Supplementary file 1 — Supplementary Information [file 41467_2026_69569_MOESM1_ESM.pdf]

Supplementary Figures

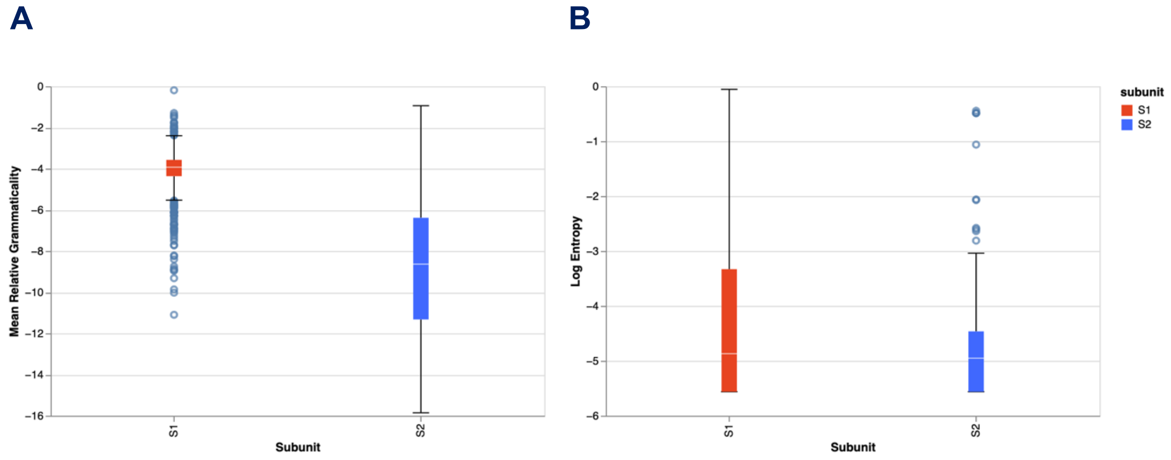

Supplementary Figure 1. **Spike subunits significantly differ in entropy and grammaticality**. (A) Boxplots for mean relative grammaticality at each site for each of the SARS-CoV-2 spike protein subunits S1 and S2. (B) Boxplots for entropy at each site for each of the SARS-CoV-2 spike protein subunits S1 and S2. Both mean relative grammaticality (p-value = 2.60e-160, two sided Mann-Whitney U test) and entropy (p-value = 1.121e-10, two sided Mann-Whitney U test) show significant differences between the subunits S1 (n=685 positions) and S2 (n=588 positions). Box plots indicate median (middle line), 25th, 75th percentile (box) and 5th and 95th percentile (whiskers) as well as outliers (single points).

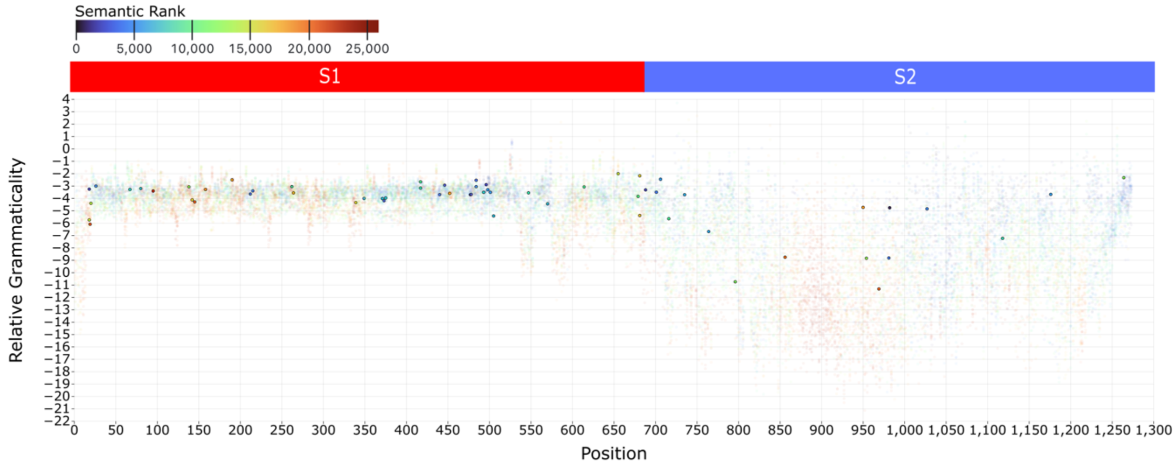

Supplementary Figure 2. **VOC mutations are more prevalent in the S1 subunit of Spike.** DMS plot with only the substitutions observed in SARS-CoV-2 VOC sequences highlighted. Most of the mutations occur in the spike protein S1 region which the model predicts has a higher likelihood of mutations, i.e., a higher grammaticality.

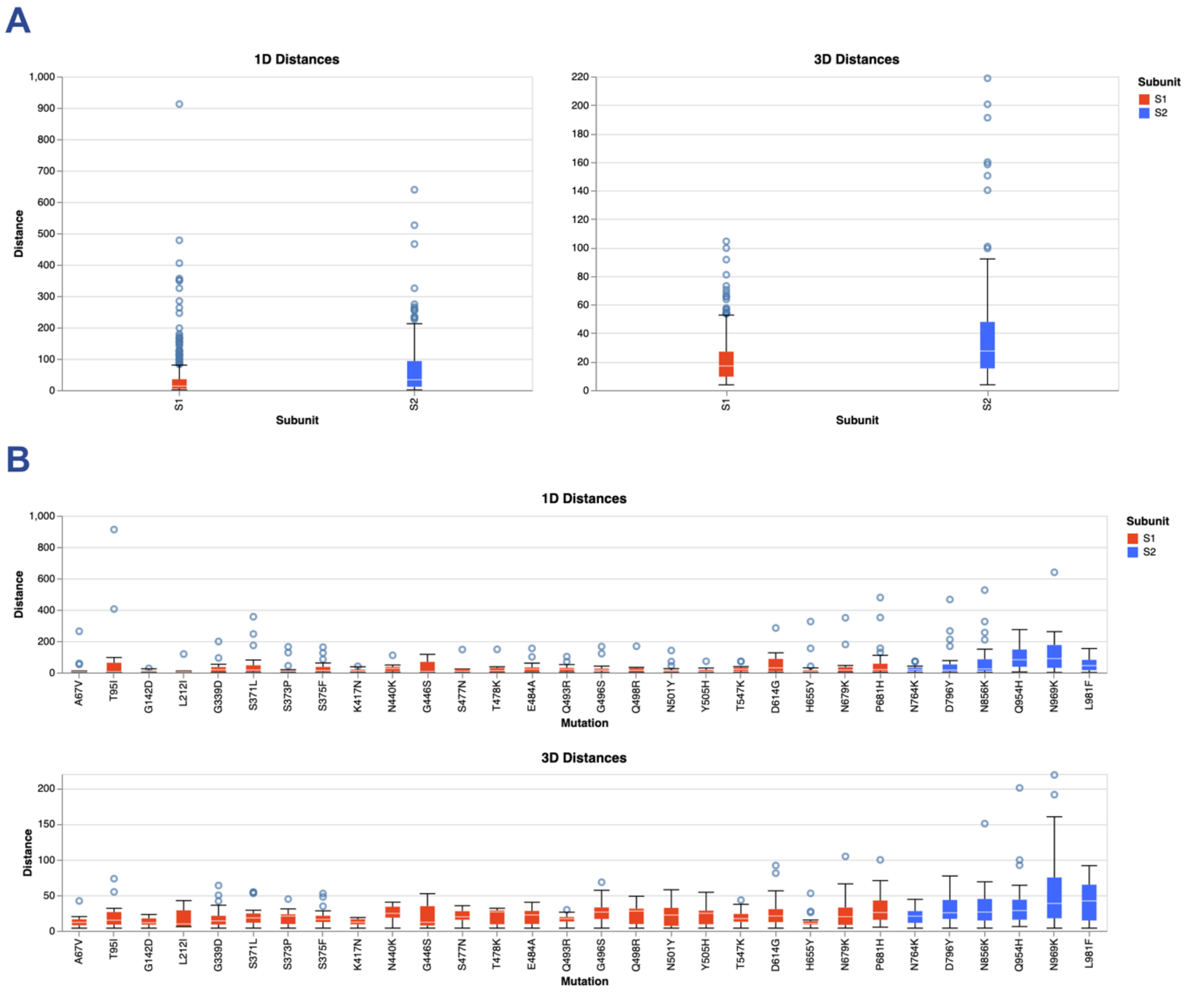

Supplementary Figure 3. **BA.1 mutations differ by their affecting distances.** (A) Boxplots showing the distribution of distances between mutations and the positions affected by mutations in each subunit. (B) Boxplots showing the distribution of sequence (1D) and protein structure (3D) distances between mutations and the positions for each mutation. Box plots indicate median (middle line), 25th, 75th percentile (box) and 5th and 95th percentile (whiskers) as well as outliers (single points)

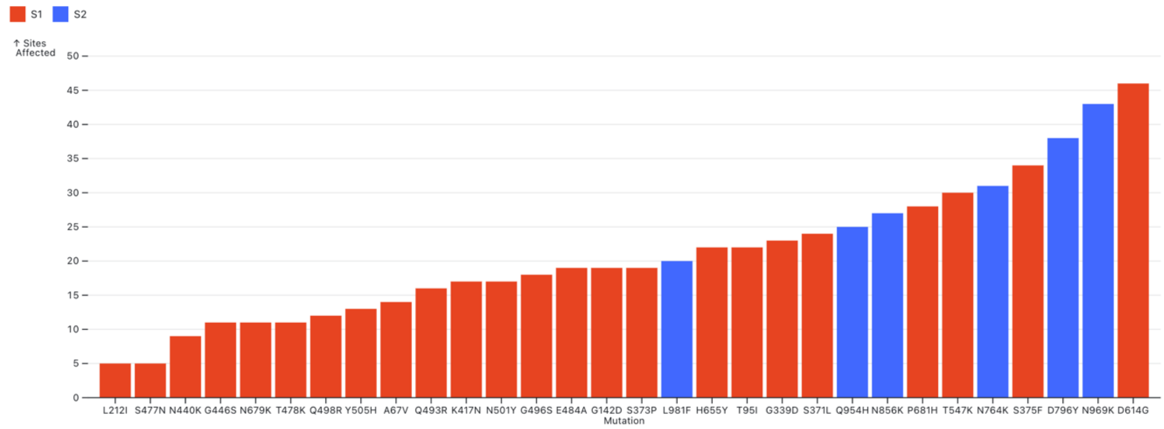

Supplementary Figure 4. **BA.1 mutations have a wide range of affected sites.** Number of sites with a significant (+/-2 deviations from mean) change in probability for each SARS-CoV-2 BA.1 reversion change.

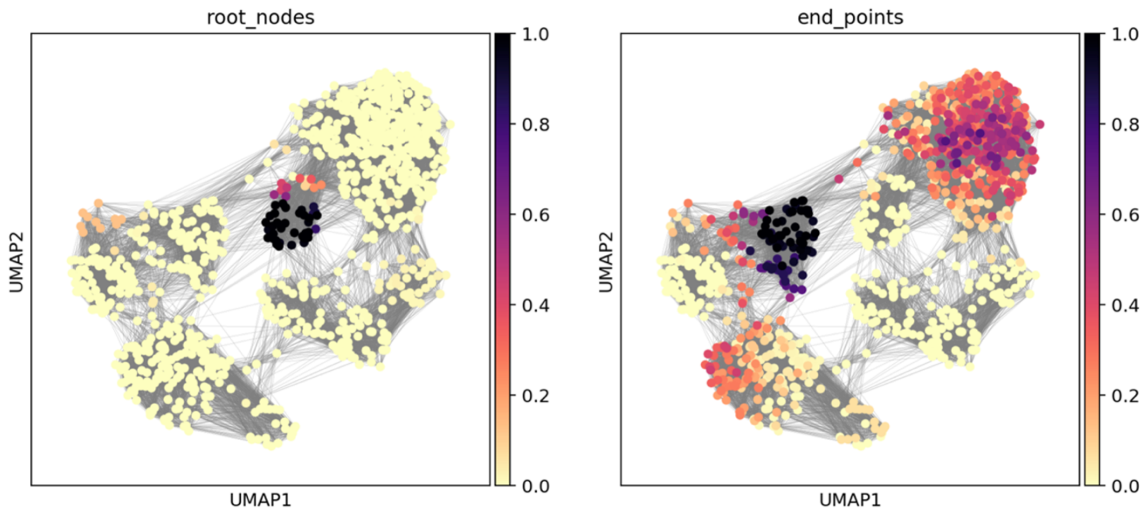

Supplementary Figure 5. **Diffusion through velocity graph identifies pre-pandemic sarbecovirus sequences as the root.** Predicted root nodes (left) and endpoints (right) identified by running Markov diffusion process over the weighted edges of the evo-velocity network. The root nodes are correctly identified as the Sarbecovirus spike sequences, with Omicron VOC sequences predicted as the end nodes. Scale represents closeness to either root or endpoints.

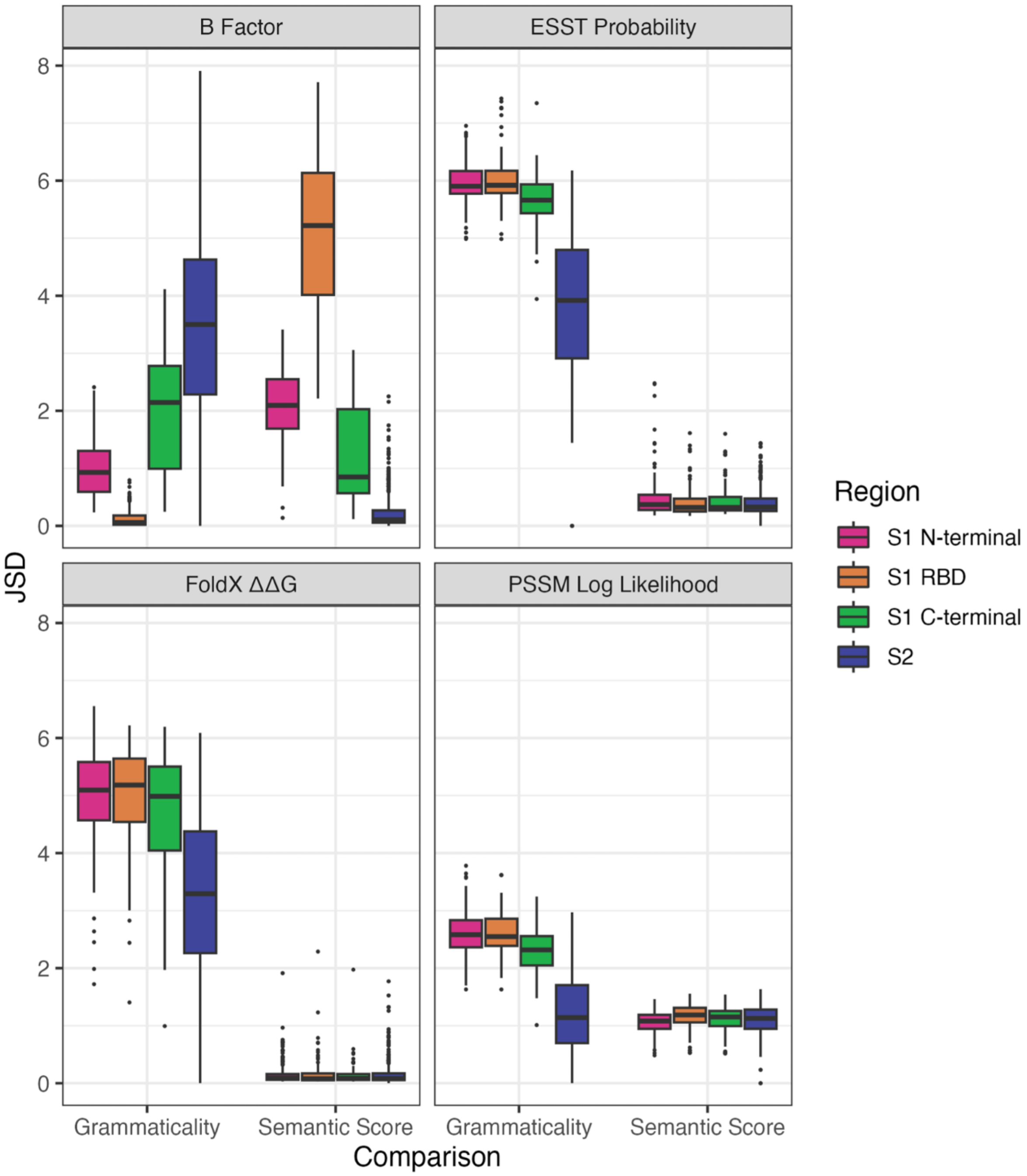

Supplementary Figure 6. **JSDs show statistically significant differences between regions in the S1 and the S2 for the grammaticality score vs other metrics.** Boxplots showing Jensen Shannon distances (JSDs) between semantic score or grammaticality and other computational metrics. JSD was calculated for every site, colours show the distribution of scores in different protein regions. Comparisons between S1 regions and S2 are all significant by Mann Whitney U test following Bonferroni correction ($p<0.05$) except for the following comparisons: FoldX ΔΔG vs semantic score; all S1 vs S2 comparisons, Log Likelihood vs semantic score; S1 C-terminal vs S2, ESST probability vs semantic score; S1 RBD vs S2, S1 C-terminal vs S2. Box plots indicate median (middle line), 25th, 75th percentile (box) and 5th and 95th percentile (whiskers) as well as outliers (single points)

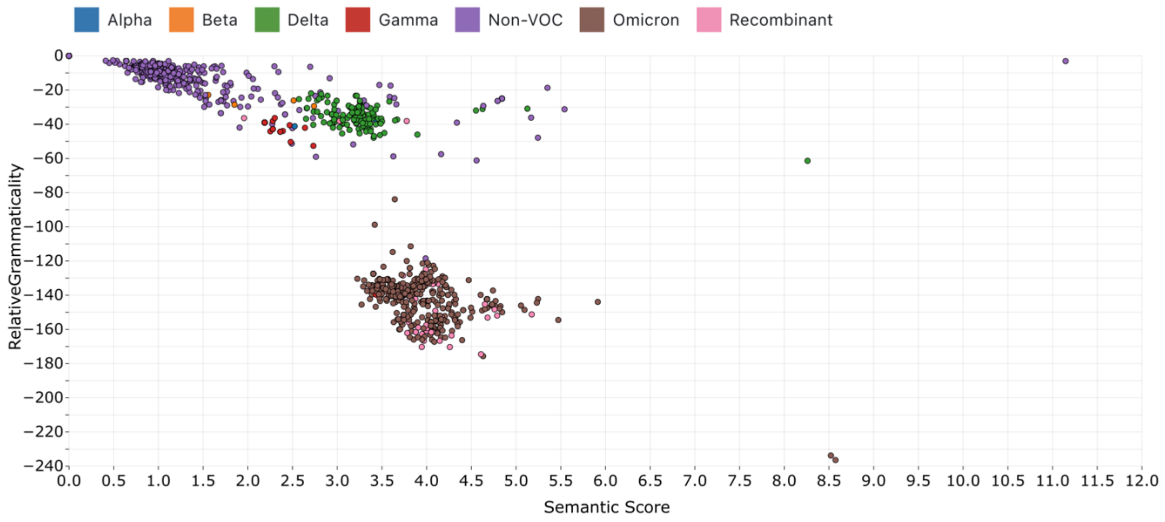

Supplementary Figure 7. **Relative grammaticality and semantic scores group related SARS-CoV-2 sequences.** SARS-CoV-2 Pango lineage representative sequences plotted by their semantic scores and relative grammaticalities.

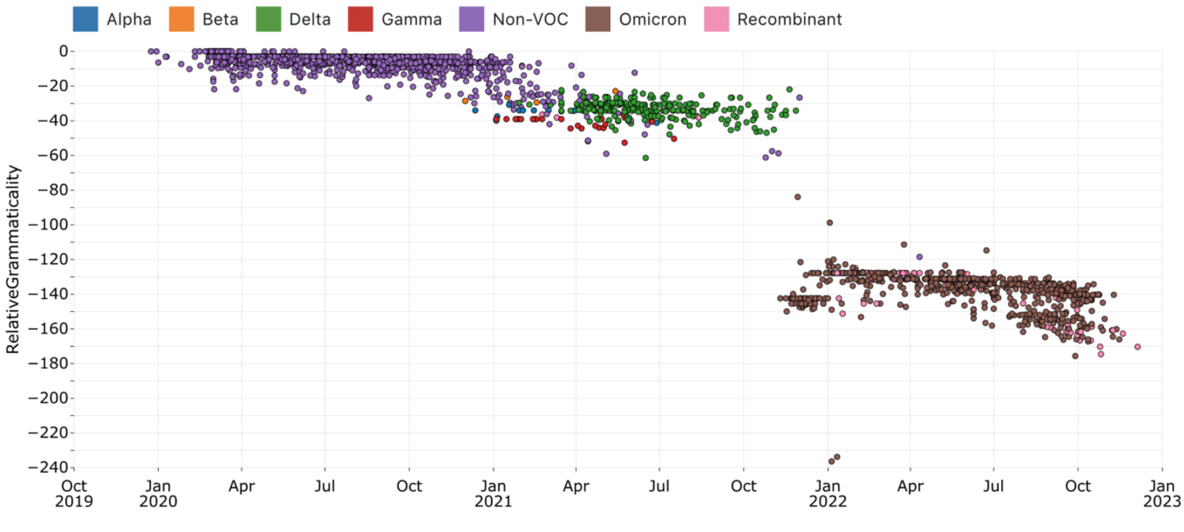

Supplementary Figure 8. **Relative grammaticality groups related SARS-CoV-2 sequences.** SARS-CoV-2 Pango lineage representative sequences plotted by their relative grammaticalities against sampling date.

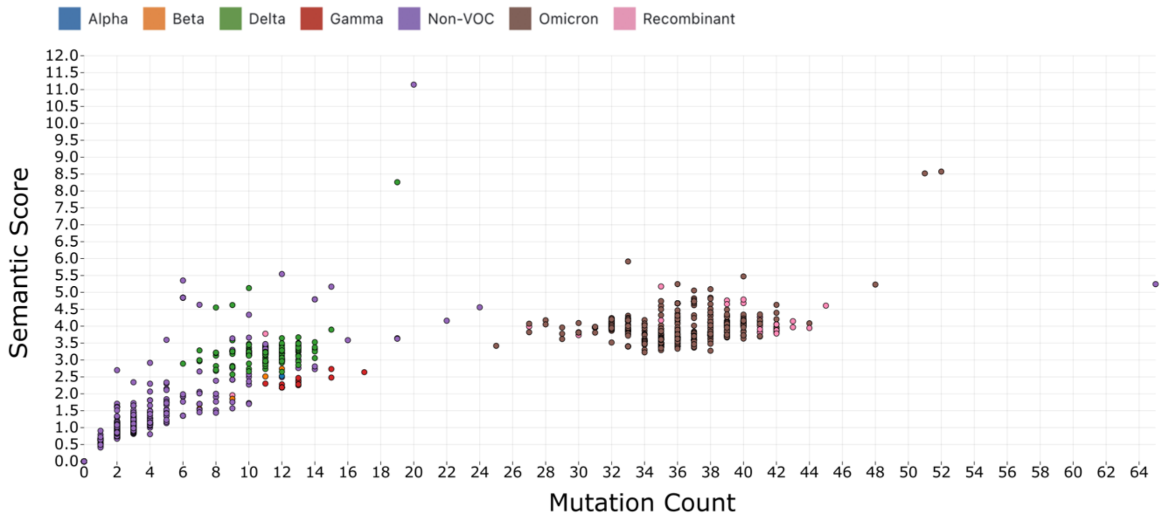

Supplementary Figure 9. **Semantic score is not equivalent to mutation count.** SARS-CoV-2 Pango lineage representative sequences plotted by their semantic score against their mutation counts. Sequences with the same mutation count have a variety of different semantic scores.

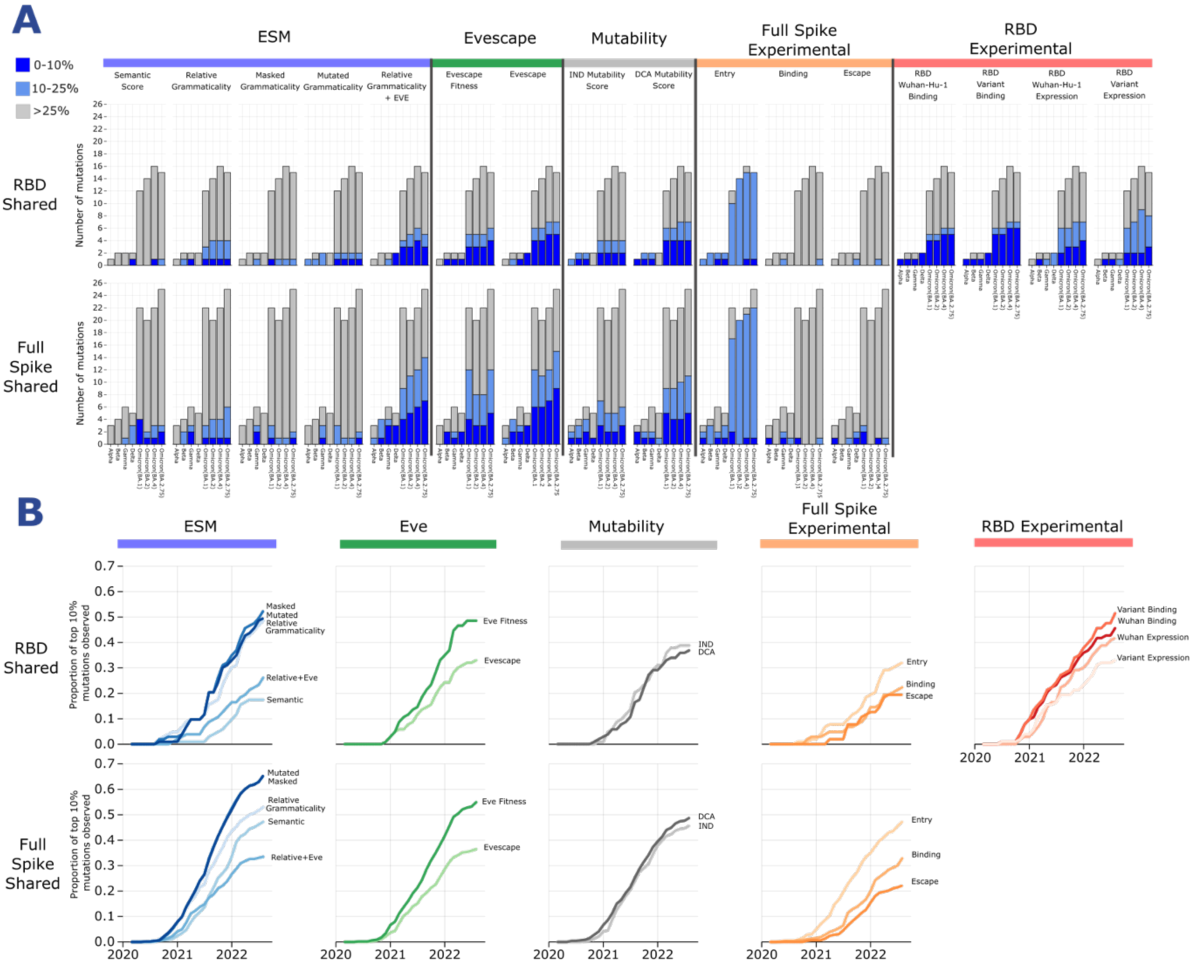

Supplementary Figure 10. **ESM metrics are comparable with other approaches.** Data from each of the mutational scans were filtered so that there is a measurement for every mutation in each dataset to allow for equivalence. The full spike mutations exclude the RBD Experimental mutations since the mutation set could only increase selection of RBD mutants given the lack of other regions in this data. (A) Barchart for each feature showing the number of mutations from variant of concern sequences present in the top 10% of shared feature predictions. The top set of bars shows the shared mutations within the RBD, while the bottom shows mutations shared across the whole of the spike protein. (B) A cumulative sum of shared DMS mutations that have appeared at least 100 times during the pandemic that appear in the top 10 % of all features.

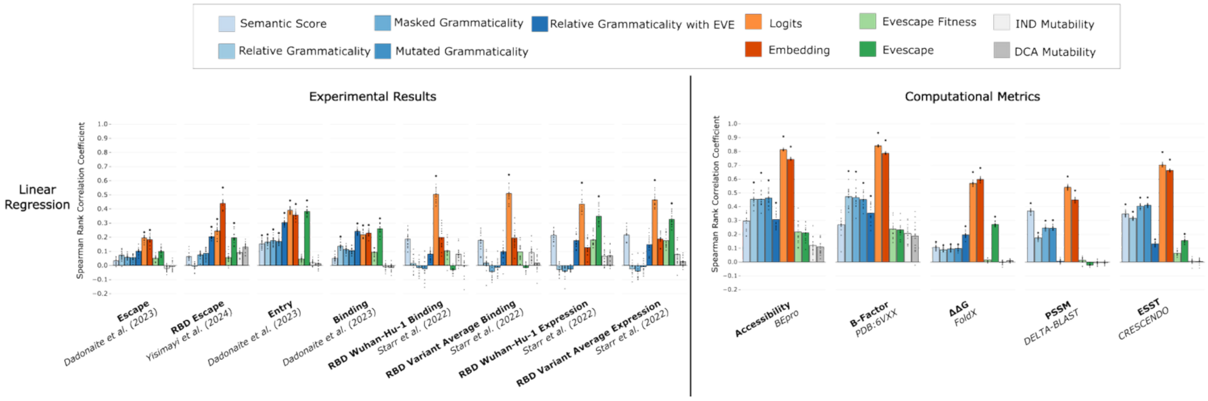

Supplementary Figure 11. **Linear regression with embeddings and logits results in improved performance over metrics.** Linear regression Spearmans rank correlation coefficients (listed in Supplementary Table 1) for each of the different feature sets. An asterisk is shown if max p-value is still significant following Bonferroni correction.

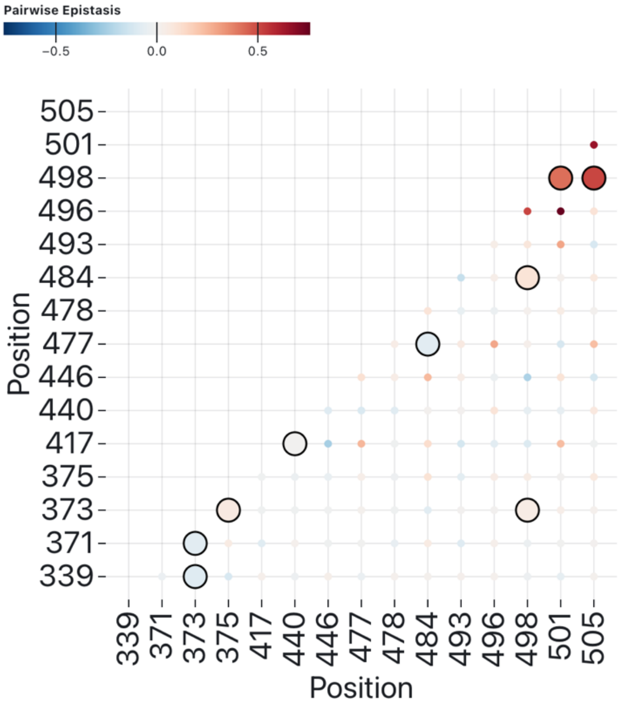

Supplementary Figure 12. **BA.1 epistatic sites identified using a mutual information approach.** Results from Moulana et al.[1] filtered by the RBD epistatic interactions identified by Innocenti et al.[2]

References

1. Moulana, A. *et al.* Compensatory epistasis maintains ACE2 affinity in SARS-CoV-2 Omicron BA.1. *Nat Commun* **13**, 7011 (2022).
2. Innocenti, G. *et al.* Real-time identification of epistatic interactions in SARS-CoV-2 from large genome collections. *Genome Biology* **25**, 228 (2024).
